# Supplementary material for: Effects of warming and nitrogen deposition on species and functional diversity of plant communities in the alpine meadow of Qinghai-Tibet Plateau
Source: PLoS One. 2025 Mar 24;20(3):e0319581. doi: 10.1371/journal.pone.0319581 (PMC11932474; doi:10.1371/journal.pone.0319581)
Supplement: S1 Table — The p-value ( < 0.05) was significant. df denotes the degree of freedom. (DOCX) [file pone.0319581.s001.docx]

**Table S1 The results of the linear mixed effect model showed the effects of warming ( W ), nitrogen deposition ( N ), and their interaction ( W × N ) on plant species diversity in the first year ( 2023 ) and the second year ( 2024 ) of the experimental treatment. The p-value ( < 0.05 ) was significant. df denotes the degree of freedom**

| **Year** | **Treatment** | **df** | **Shannon-wiener’s diversity index** | | **Species richness index** | | **Species evenness index** | | **Important value of grasses** | | **Important value of sedges** | | **Important value of forb** | |
| --- | --- | --- | --- | --- | --- | --- | --- | --- | --- | --- | --- | --- | --- | --- |
|  |  |  | **F** | **P value** | **F** | **P value** | **F** | **P value** | **F** | **P value** | **F** | **P value** | **F** | **P value** |
| 2023 | W | 3 | 12.03 | 0 | 3.41 | 0.13 | 3.67 | 0.10 | 48.31 | 0 | 3.30 | 0.03 | 2.97 | 0.04 |
|  | N | 2 | 1.08 | 0.23 | 0.99 | 0.40 | 3.47 | 0.11 | 31.31 | 0 | 1.20 | 0.52 | 0.78 | 0.48 |
|  | W×N | 6 | 0.51 | 0.54 | 0.35 | 0.96 | 1.33 | 0.29 | 5.90 | 0.04 | 2.37 | 0.04 | 1.32 | 0.27 |
| 2024 | W | 3 | 19.19 | 0 | 3.66 | 0.04 | 2.55 | 0.37 | 90.71 | 0 | 55.67 | 0 | 14.93 | 0 |
|  | N | 2 | 15.03 | 0 | 1.09 | 0.45 | 1.86 | 0.17 | 36.28 | 0 | 28.45 | 0 | 3.04 | 0.06 |
|  | W×N | 6 | 8.90 | 0.01 | 0.46 | 0.84 | 0.20 | 0.99 | 3.04 | 0.02 | 5.81 | 0.02 | 0.15 | 0.87 |
| Overall | Y | 1 | 1.04 | 0.67 | 0.09 | 0.77 | 1.28 | 0.50 | 1.21 | 0.57 | 1.21 | 0.61 | 1.03 | 0.68 |
|  | W | 3 | 20.92 | 0 | 7.68 | 0 | 1.99 | 0.11 | 58.54 | 0 | 28.01 | 0 | 48.82 | 0 |
|  | N | 2 | 19.23 | 0 | 3.11 | 0.07 | 1.16 | 0.60 | 34.36 | 0 | 17.11 | 0 | 2.95 | 0.06 |
|  | W×N | 6 | 5.57 | 0.03 | 0.09 | 0.74 | 2.01 | 0.10 | 4.35 | 0.01 | 2.30 | 0.17 | 0.50 | 0.47 |
|  | Y×N | 2 | 1.74 | 0.18 | 0.10 | 0.90 | 1.52 | 0.23 | 1.47 | 0.24 | 1.22 | 0.30 | 0.64 | 0.53 |
|  | Y×W | 3 | 5.28 | 0.08 | 0.58 | 0.97 | 0.59 | 0.62 | 2.44 | 0.44 | 4.55 | 0.07 | 0.94 | 0.57 |
|  | Y×W×N | 6 | 0.71 | 0.64 | 0.09 | 0.99 | 0.52 | 0.69 | 0.97 | 0.45 | 1.46 | 0.51 | 0.63 | 0.70 |
