# Supplementary material for: Effects of warming and nitrogen deposition on species and functional diversity of plant communities in the alpine meadow of Qinghai-Tibet Plateau
Source: PLoS One. 2025 Mar 24;20(3):e0319581. doi: 10.1371/journal.pone.0319581 (PMC11932474; doi:10.1371/journal.pone.0319581)
Supplement: S2 Table — The p-value ( < 0.05) was significant. df denotes the degree of freedom. (DOCX) [file pone.0319581.s002.docx]

**Table S2 The results of the linear mixed effect model showed the effects of warming ( W ), nitrogen deposition ( N ), and their interaction ( W × N ) on plant functional diversity in the first year ( 2023 ) and the second year ( 2024 ) of the experimental treatment. The p-value ( < 0.05 ) was significant. df denotes the degree of freedom**

| **Year** | **Treatment** | **df** | **Functional richness index** | | **Functional evenness index** | | **Functional divergence index** | | **Functional dispersion index** | | **Rao’s quadratic entropy index** | |
| --- | --- | --- | --- | --- | --- | --- | --- | --- | --- | --- | --- | --- |
|  |  |  | **F** | **P value** | **F** | **P value** | **F** | **P value** | **F** | **P value** | **F** | **P value** |
| 2023 | W | 3 | 7.28 | 0 | 8.87 | 0 | 1.19 | 0.11 | 60.77 | 0 | 78.36 | 0 |
|  | N | 2 | 0.44 | 0.51 | 2.42 | 0.10 | 0.27 | 0.77 | 76.20 | 0 | 113.63 | 0 |
|  | W×N | 6 | 3.96 | 0.04 | 1.67 | 0.24 | 1.04 | 0.40 | 4.14 | 0.03 | 5.48 | 0.02 |
| 2024 | W | 3 | 9.03 | 0 | 7.88 | 0 | 1.64 | 0.14 | 49.09 | 0 | 44.73 | 0 |
|  | N | 2 | 5.72 | 0.03 | 1.86 | 0.22 | 1.81 | 0.25 | 24.87 | 0 | 22.11 | 0 |
|  | W×N | 6 | 6.71 | 0.004 | 4.11 | 0.03 | 1.98 | 0.15 | 2.93 | 0.04 | 2.63 | 0.04 |
| Overall | Y | 1 | 2.74 | 0.06 | 2.72 | 0.07 | 1.76 | 0.24 | 2.11 | 0.10 | 1.91 | 0.44 |
|  | W | 3 | 7.91 | 0 | 9.61 | 0 | 1.37 | 0.37 | 98.76 | 0 | 95.07 | 0 |
|  | N | 2 | 4.48 | 0.04 | 1.21 | 0.31 | 1.20 | 0.28 | 72.74 | 0 | 69.67 | 0 |
|  | W×N | 6 | 5.31 | 0.01 | 5.38 | 0.004 | 1.84 | 0.44 | 1.48 | 0.10 | 3.36 | 0.03 |
|  | Y×N | 2 | 1.62 | 0.15 | 1.48 | 0.24 | 1.78 | 0.44 | 1.46 | 0.06 | 2.53 | 0.40 |
|  | Y×W | 3 | 2.18 | 0.08 | 1.32 | 0.32 | 1.57 | 0.07 | 1.16 | 0.30 | 1.91 | 0.30 |
|  | Y×W×N | 6 | 1.92 | 0.13 | 2.23 | 0.07 | 1.13 | 0.36 | 2.07 | 0.07 | 2.07 | 0.06 |
