# Supplementary material for: Effects of warming and nitrogen deposition on species and functional diversity of plant communities in the alpine meadow of Qinghai-Tibet Plateau
Source: PLoS One. 2025 Mar 24;20(3):e0319581. doi: 10.1371/journal.pone.0319581 (PMC11932474; doi:10.1371/journal.pone.0319581)
Supplement: S3 Table — The p-value ( < 0.05) was significant. df denotes the degree of freedom. (DOCX) [file pone.0319581.s003.docx]

**Table S3 The results of the linear mixed effect model showed the effects of warming ( W ), nitrogen deposition ( N ), and their interaction ( W × N ) on the weighted average of plant community functional traits in the first year ( 2023 ) and the second year ( 2024 ) of the experimental treatment. The p-value (< 0.05) was significant. df denotes the degree of freedom**

| **Year** | **Treatment** | **DF** | **CWM_VH** | | **CWM_LA** | | **CWM_LL** | | **CWM_LW** | | **CWM_LDMC** | | **CWM_SLA** | | **CWM_ LPC** | | **CWM_LNC** | | | **CWM_LCC** | |
| --- | --- | --- | --- | --- | --- | --- | --- | --- | --- | --- | --- | --- | --- | --- | --- | --- | --- | --- | --- | --- | --- |
|  |  |  | **F** | **P** | **F** | **P** | **F** | **P** | **F** | **P** | **F** | **P** | **F** | **P** | **F** | **P** | **F** | **P** | | **F** | **P** |
| 2023 | W | 3 | 21.22 | 0 | 18.89 | 0 | 12.71 | 0 | 2.91 | 0.05 | 23.11 | 0 | 3.95 | 0.01 | 0.75 | 0.41 | 100.13 | 0 | | 2.43 | 0.13 |
|  | N | 2 | 2.18 | 0.13 | 24.03 | 0 | 9.88 | 0 | 1.03 | 0.37 | 12.62 | 0 | 0.29 | 0.75 | 1.25 | 0.39 | 100.83 | | 0 | 0.06 | 0.58 |
|  | W×N | 6 | 1.43 | 0.23 | 1.45 | 0.22 | 0.31 | 0.93 | 1.82 | 0.12 | 4.36 | 0.002 | 2.75 | 0.02 | 1.30 | 0.44 | 7.55 | 0 | | 1.63 | 0.32 |
| 2024 | W | 3 | 39.74 | 0 | 30.53 | 0 | 29.93 | 0 | 2.56 | 0.07 | 16.72 | 0.01 | 13.64 | 0 | 1.94 | 0.13 | 82.45 | 0 | | 89.92 | 0 |
|  | N | 2 | 9.21 | 0.02 | 60.35 | 0 | 22.16 | 0 | 2.81 | 0.06 | 56.73 | 0 | 86.48 | 0 | 2.33 | 0.15 | 46.71 | 0 | | 53.28 | 0 |
|  | W×N | 6 | 12.52 | 0 | 8.62 | 0 | 17.91 | 0 | 1.25 | 0.33 | 5.56 | 0 | 81.40 | 0 | 1.48 | 0.44 | 96.30 | 0 | | 44.10 | 0 |
| Overall | Y | 1 | 2.62 | 0.27 | 2.42 | 0.12 | 1.21 | 0.45 | 0.60 | 0.51 | 0.52 | 0.47 | 1.80 | 0.25 | 1.42 | 0.31 | 1.48 | 0.28 | | 1.81 | 0.35 |
|  | W | 3 | 57.29 | 0 | 50.92 | 0 | 27.82 | 0 | 1.25 | 0.57 | 24.05 | 0 | 29.59 | 0 | 1.73 | 0.35 | 29.89 | 0 | | 11.54 | 0 |
|  | N | 2 | 5.14 | 0.03 | 25.89 | 0 | 27.94 | 0 | 1.33 | 0.41 | 19.60 | 0 | 23.58 | 0 | 2.18 | 0.44 | 37.52 | 0 | | 27.91 | 0 |
|  | Y×W | 6 | 1.72 | 0.30 | 1.03 | 0.47 | 1.03 | 0.48 | 1.63 | 0.29 | 1.64 | 0.29 | 1.44 | 0.41 | 1.87 | 0.28 | 1.40 | 0.29 | | 1.63 | 0.34 |
|  | Y×N | 2 | 1.27 | 0.47 | 1.69 | 0.35 | 1.29 | 0.41 | 1.34 | 0.39 | 1.01 | 0.28 | 1.87 | 0.36 | 1.67 | 0.24 | 1.27 | 0.37 | | 1.52 | 0.25 |
|  | W×N | 3 | 7.82 | 0 | 8.27 | 0 | 12.97 | 0 | 1.49 | 0.34 | 13.35 | 0.04 | 13.19 | 0.03 | 1.21 | 0.35 | 33.21 | 0 | | 21.62 | 0 |
|  | Y×W×N | 6 | 1.47 | 0.36 | 1.45 | 0.28 | 1.18 | 0.16 | 1.61 | 0.36 | 1.54 | 0.28 | 1.03 | 0.34 | 1.37 | 0.29 | 1.23 | 0.19 | | 1.37 | 0.43 |

Note: CWM_VH, CWM_LA, CWM_LL, CWM_LW, CWM_LDMC, CWM_SLA, CWM_ LPC, CWM_LNC,CWM_LCC are expressed as weighted mean of plant height, weighted mean of leaf area, weighted mean of leaf length, weighted mean of leaf width, weighted mean of leaf weight, respectively. Weighted mean of specific leaf area, weighted mean of leaf phosphorus, weighted mean of leaf nitrogen, weighted mean of leaf carbon
