# Supplementary material for: Effects of warming and nitrogen deposition on species and functional diversity of plant communities in the alpine meadow of Qinghai-Tibet Plateau
Source: PLoS One. 2025 Mar 24;20(3):e0319581. doi: 10.1371/journal.pone.0319581 (PMC11932474; doi:10.1371/journal.pone.0319581)
Supplement: Attachment — Submitted filename: Submissions Needing Revision.doc [file pone.0319581.s005.doc]

**Dear editors and reviewers:**

Thank you for your letter and for the reviewers’ comments concerning our manuscript entitled “**Soil Nutrients Explain Plant Community Species Diversity Changes Better Than Functional Traits Under Short-Term Warming and Nitrogen Deposition in Alpine Meadows**” (Manuscript ID. PONE-D-24-38030). Those comments are all valuable and very helpful for revising and improving our paper, as well as the important guiding significance to our research. We have studied comments carefully and have made corrections which we hope meet with approval. The main corrections in the paper and the responses to the reviewers’ comments are as follows:

**Reviewer1:**

**Question 1: This study only explored the impact of warming and nitrogen addition on plant species diversity, but did not discuss the changes in plant phylogenetic diversity. Some previous studies have shown that external disturbances such as warming and nitrogen addition have different effects on plant species and phylogenetic diversity (e.g., doi:10.1016/j.ecoleng.2021.106331; doi:10.1016/j.ecolind.2021.108355; doi:10.1016/j.gecco.2023.e02464; doi:10.3389/fevo.2023.1126651; doi:10.3390/plants12173017; doi:10.1016/j.scitotenv.2023.168878). Therefore, it is recommended that the author add relevant discussions in the discussion section.**

**Response:** **We recognize that phylogenetic diversity in plant communities is an important dimension of biodiversity. While this study focused primarily on species diversity and functional traits, we agree that phylogenetic diversity is also a key aspect that contributes to a deeper understanding of how warmth and nitrogen deposition affect plant communities. However, due to the diversity of development, this study did not directly measure system refer to the latest research (such as 10.1016 / j. coleng. 2021.106331), and has set up a file in the manuscript clearly points out the limitations, and emphasis on future research should consider this on the one hand, to provide a more comprehensive understanding of the responses of a community. Modified as follows (L634-L641) :**

**-**In conclusion, as an important part of biodiversity, plant diversity plays an important role in maintaining the stability of grassland ecosystems. Phylogenetic diversity can reflect the ecological process of plant community composition and plays an important role in revealing the maintenance mechanism of biodiversity (Sun et al. 2021). Therefore, exploring the effects of warming and nitrogen deposition on plant phylogenetic diversity in future studies can better understand the corresponding mechanisms of plant diversity on climate change in grassland ecosystems.

**Question 2: In addition to conventional soil physicochemical properties, soil microbial community diversity is closely related to plant diversity (e.g., doi:10.1016/j.scitotenv.2023.168878; DOI: 10.1016/j.gecco.2022.e02306). Therefore, it is recommended that the author add discussions on the regulation of plant diversity by soil microbial diversity**

**Response: We agree with reviewer #1 that soil microbial community diversity is closely related to plant diversity. While this study focused primarily on the effects of warmth and nitrogen additions on plant communities and soil physicochemical properties, we recognize that microbial communities play an important role in understanding plant diversity in alpine meadows. In response to this, we have added to the discussion section of the revised manuscript a discussion on the potential role of soil microbial community diversity in regulating plant diversity. We cite studies (DOI: DOI: 10.1016 / j.Geecco. 202.e02306) highlighting the effects of soil microorganisms on plant species diversity and functional status under climate change. We believe that these additions make the manuscript more complete. Thanks again to reviewer #1 for their valuable suggestions, which greatly enhanced the quality of the manuscript. Modified as follows (L676-L694) :**

**-**The results of this study also showed that soil C/N ratio was also an important factor affecting the species and functional diversity of plant communities. The study of 84 grasslands across six continents across a wide range of climatic gradients also found that plant diversity was positively correlated with soil C/N ratio(Spohn et al. 2023). This is mainly due to the low nutritional value of organic matter with a high carbon-nitrogen ratio to microorganisms and slow decomposition(Cotrufo et al. 2013). Relatively scarce nitrogen resources may affect the competition between plants, thus affecting the diversity of plant communities. At the same time, warming and nitrogen deposition will affect the species and functional diversity of plant communities, and the diversity of plant species increases the cost of soil microbial decomposition(Han et al. 2022). Very diverse organic compounds from different plant communities may decompose more slowly than less diverse organic matter from less diverse communities (El Moujahid et al. 2017, Lehmann et al. 2020). Soil factors determine which species or functional traits in plant communities will be retained, and this variability in plant species ' adaptation to the environment affects competition between communities and affects plant community structure(Wang et al. 2022b). Therefore, a better understanding of the relationship between plant community species diversity and soil physical and chemical properties will help to understand the overall function of grassland ecosystems better.

**We believe that these revisions have significantly improved the manuscript and have addressed all the issues raised. Thank you again for your valuable feedback.**

**Reviewer2:**

**Question 1: The manuscript presents an experimental field study conducted in an alpine meadow in Qinghai-Tibet Plateau. In this study, researchers experimentally simulated the different levels of warming and nitrogen deposition and studied their effect on changes in plant community composition. They focused on the influence of functional traits and soil physicochemical properties on species richness, diversity and evenness under altering warming and nitrogen deposition. The study is interesting although it is conducted only on one alpine meadow, which limits authors to make some more general conclusions. It could be also interesting to observe the effects for longer time and evaluate temporal changes. I understand that multisite and temporal studies are not always possible to do, but it should be at least stated in discussion as study limitations. And this should be also reflected in the title of the paper. You cannot writhe “in Alpine meadows”, when your study was conducted only on one unique site. The topic of the study seems to be nowadays quite popular, but also very often studied and thus not very novel. Nevertheless, it is quite useful to get such information from different places and I also like the comparison of the influence of traits and soil properties. I think, it is quite interesting point of view.**

**In general, the topic is interesting, and manuscript is written well. Nevertheless, there are some things, I am not very happy with. Especially, the methods should be more appropriate – there are many missing details about experimental design, measurements and formulas. Some conceptual figure showing the experimental design could be helpful. In the whole manuscript, the names of variables and other terms are not unified and thus, it is very difficult to follow the text. There is a chaos in used formulas and the indexes and how you call them through the text. A part of the results is not presented in a well-arranged way. Some results are presented only in the text without any figure or table support, especially one factor results (in figures are results only for interactions, and values of statistical test with degrees of freedom and p values are missing almost everywhere). Arrangements of these values into a table could be helpful. There are more typing errors in the text, which I did not corrected.**

**Because the text does not have nor line numbers nor page numbers, it is very difficult to refer to something specific (and I have some suggestions). Thus, I am using at least the pdf page numbering, when necessary.**

**Response: We greatly appreciate the reviewers' detailed and constructive feedback, which is of great value in improving the clarity and rigor of our manuscripts. The corresponding line numbers have been added in the revised draft. The following are our specific responses to the comments:**

**Question 2: Abstract“Additionally, Short-term nitrogen deposition affects plant community structure but has no significant impact on species diversity.” – It is not clear for me, what is the difference between community structure and diversity. It is not very clear nor from the further text. For me, community structure could be represented either by species richness or by diversity. It could also be the functional diversity or at least the proportion of functional groups. In any case, it is necessary to specify it more or write directly what do you mean.**

**Response: Thanks for your suggestion, the summary's mention that "short-term nitrogen deposition affects plant community structure but has no significant effect on species diversity" is really confusing. According to the suggestions of reviewers, the overall framework and results of the article are reorganized. Therefore, the summary part is modified. Modify as follows (L5-L24) :**

**-**Plant species and functional diversity play an important role in the stability and sustainability of grassland ecosystems. However, the changes and mechanisms of plant species and functional diversity under warming and nitrogen deposition are still unclear. In this study, we investigated the plant and soil characteristics of alpine meadows on the Qinghai-Tibet Plateau to explore the changes in species and functional diversity of plant communities under warming and nitrogen deposition, as well as their interrelationships and key determinants. The results showed that warming, nitrogen deposition, and their interactions had significant effects on plant species diversity ( plant Shannon-Wiener index ) and functional diversity ( functional richness index, functional differentiation index, functional dispersion, and Rao's secondary entropy index ). With the increase of warming and nitrogen deposition, the Shannon-Wiener index of plants increased first and then decreased. The plant functional richness index, functional diversity index, functional dispersion index, and Rao's secondary entropy index showed a decreasing trend. At the same time, with the increase in temperature and nitrogen deposition, the relationship between plant species diversity index and functional diversity index in the alpine meadow of Qinghai-Tibet Plateau gradually weakened. Redundancy analysis and structural equation modeling showed that both warming and nitrogen deposition had significant negative effects on the plant species diversity index and plant functional diversity index. Plant factors ( Grasses importance value, leaf nitrogen weighted mean, specific leaf area-weighted mean, leaf area-weighted mean, and leaf weight weighted mean ) and soil environmental factors ( soil total nitrogen and soil carbon-nitrogen ratio ) directly or indirectly affect plant community diversity under warming and nitrogen deposition.

**Question 3: Introduction**

**Page 10: I do not like very much the first paragraph of the introduction, where you are listed everything together without going in detail (especially the part about functional group and nitrogen deposition). I prefer to describe it separately in individual paragraph with more details.**

**Response:** **Thanks for your suggestion, rereading this paragraph does feel that the logic is not clear enough, and the impact of nitrogen deposition on plant diversity is only emphasized in this part. Therefore, read relevant literature, supplement relevant views, divide the paragraph into two paragraphs to make the structure clearer, and modify it as follows (L32-L62) :**

**-**Global surface temperatures are projected to rise by approximately 4°C by 2100 (Wang et al. 2020). Simultaneously, anthropogenic nitrogen emissions and deposition are increasing rapidly on a worldwide scale(Kwaku et al. 2021). In the past few decades, the combined effects of climate change factors such as temperature rise and nitrogen deposition have led to the degradation of the function and structure of grassland ecosystems, resulting in accelerated desertification, loss of biodiversity, and changes in carbon balance(Chen et al. 2022). The loss of biodiversity not only means the extinction of species but also poses a threat to food security, social stability, and human survival through the food chain and food web.

Plant community diversity is an important part of biodiversity(Zha et al. 2022). Plant species diversity reflects the complex relationship between biology, environment, and biological resource richness(Katovai et al. 2012). Research has shown that global warming significantly reduces plant species richness and diversity in terrestrial ecosystems (Yang et al. 2011). Different plant functional groups respond differently to climate warming (Fay et al. 2011). The effects of nitrogen deposition on plant species diversity vary depending on the nitrogen use efficiency and adaptability of the plant community (Roth et al. 2015, Zhang et al. 2015, Kwaku et al. 2021). In the study of temperate grassland and semi-arid grassland, it was found that plant species richness showed a downward trend under nitrogen deposition (Zhang et al. 2014, He et al. 2016). With the increase in temperature and nitrogen deposition, the number of vascular plants is generally increasing, which may be because temperature and nutrients are the main limiting factors of alpine ecosystems(Boutin et al. 2017). Plant functional diversity takes into account the redundant and complementary functions within the plant community, as well as the response of functional traits to environmental stress or disturbance(Li et al. 2024). Species diversity and functional diversity are key determinants of grassland ecosystem stability (Petchey and Gaston 2006, Dunck et al. 2016). Therefore, exploring the feedback mechanism of plant community diversity on climate warming and nitrogen deposition is of great scientific value and practical guiding significance for scientifically understanding the maintenance mechanism of grassland ecosystem diversity on the Qinghai-Tibet Plateau under climate change and guiding grassland management.

**Question 4: Page 11:
What do you mean by “physiological traits”? Because in following text, you describe both morphological (SLA, leaf nitrogen concentration) and eco-physiological (photosynthetic rate) traits.
“Beyond leaf economics traits” – photosynthetic rate is not LES trait.**

**Response: Thanks for your suggestion, after reading the relevant literature, it is indeed found that the photosynthetic rate is not an economic trait of leaves, and this part has been modified. Modify as follows (L73-L75) :**

**-** Studies have shown that nitrogen enrichment enhances specific leaf area, and leaf nitrogen concentration in plant species (Eskelinen and Harrison 2015, La Pierre and Smith 2015).

**Question 5: Page 12:**

**“Studies on the Qinghai-Tibet Plateau indicate that soil organic matter, available nitrogen, and phosphorus significantly affect plant diversity in alpine meadows [22].” – You are writing studies, but then you are citing only one study although it is not metanalysis. The same for more cases throughout the whole text (including discussion).**

**Response:Thank you for your suggestion. This paragraph mainly focuses on the discussion that the physical and chemical properties of soil have a significant impact on plant diversity, but the conclusions reached by different studies are inconsistent. Therefore, it is necessary to explore the effects of soil physicochemical properties on plant diversity under climate warming and nitrogen deposition. Read the relevant literature and rearrange the section. Modified as follows (L93-L106) :**

**-**In temperate grassland, soil pH, total nitrogen, and nitrogen-phosphorus ratio are the key factors to explain plant functional diversity(Rodríguez et al. 2023). Studies have also shown that soil nitrogen content in the short term and soil phosphorus content in the long term affect plant species richness in the long term(Anic et al. 2010). Studies on the Qinghai-Tibet Plateau have shown that soil organic matter, available nitrogen, and phosphorus significantly affect plant diversity in alpine meadows (Wang et al. 2007). In the semi-arid region of the Qinghai-Tibet Plateau, the depth of permafrost active layer and soil water content are important factors affecting the distribution of plant communities (Wang et al. 2012). Combined with the above, the key soil physical and chemical factors affecting the diversity of plant communities in different research areas are different. Therefore, it is necessary to clarify the relationship between plant community diversity and soil physical and chemical properties in alpine meadows on the Qinghai-Tibet Plateau, which will help to understand the overall function of grassland ecosystems better.

**Question 6: Materials and Methods**

**Page 14:**

**Elymus nutans and Poa annua are not forbs but grasses. And forbs (or grasses) are not family, but functional group. And is it Poa annua or P. pratensis. In Fig. 2, you have only P. pratensis.**

**50 x 50 m2 does not make sense. It is either 50 x 50 m or 2500 m2.**

**Response: Thank you for your advice. Through the suggestions of the reviewers, I found that the materials and methods were not clear enough. Therefore, revise this section again. 50x50m2 has been changed to 50x50m.**

**Question 7: Page 15:**

**I would like more details about soil and air temperature measurement. In which distance from the soil surface was the temperature measured ? How frequently the sensors measured the temperature? What does “CK” mean?**

**Fig. 1:**

**a) and b) show changes in temperature for individual warming treatment. But before, you stated that you had the combination of plots with simulated warming with nitrogen deposition. Does it mean that these values are averages for W0N0, W0N16 and, W0N32, and so as? It should be stated in methodology.**

**Why there are axis labels in Chinese language?**

**The colour of columns of different treatment in c) and d) could be the same as line in a) and b). It would increase the readability of the figure.**

**For c) and d), it should be stated in the caption what do the letters above the columns mean? I expected that it is the result of Tukey test, but it should be stated in the caption of figure and also, this test should be described in methodology appropriately (what was compared with what).**

**Response:Thank you for your advice, but there is not enough detail in the temperature measurement section. In order to more intuitively understand the height of the temperature measurement from the ground, the temperature increase range and the measurement time, the table is modified. Modify as follows (L147-L160) :**

**-**Studies have shown that the Tibetan Plateau is expected to face an additional warming of up to 2.0℃ by 2035 and an additional warming of 4.9℃ by 2100 (Liu et al. 2009). In order to simulate different levels of warming and adjust the diameter of the top and bottom of OTC, four temperature treatments (no warming and three warming treatments ) were selected: no warming ( W0 ), warming 1 ( W1, top diameter of 1.5m, bottom diameter of 1.95m ), warming 2 ( W2, top diameter of 1m, bottom diameter of 1.45m ), warming 3 ( W3, top diameter of 0.5m, bottom diameter of 0.95m ). The height of the open-top growth chamber (OTC) was 40 cm. Soil and air temperatures are continuously measured from June to October using the HOBOS-TMB-M006 temperature sensor (Temperature Smart Sensor, HOBO, USA). The warming effect is shown in Table 1.

Table 1 Specifications and effects of heating devices

| Heating Gradient （℃） | Top Diameter （m） | Bottom Diameter （m） | Height （m） | Temperature（℃） | |
| --- | --- | --- | --- | --- | --- |
|  |  |  |  | Air temperature at 10cm above the ground | soil temperature at 5cm below the ground surface |
| W1 | 1.5 | 1.94 | 0.4 | 0.47 | 0.61 |
| W2 | 1 | 1.45 | 0.4 | 0.92 | 1.09 |
| W3 | 0.5 | 0.95 | 0.4 | 1.44 | 1.95 |

Note: The warming effect of W1, W2, and W3 is compared with that of W0 (no warming). The data in the table is the mean for June to October.

**Thank you for your suggestion, "CK" is not warming and not adding nitrogen treatment, is a contrast. But I found that the need for annotations was confusing when reading the text. Therefore, delete this section. Modified as follows (L161-171):**

**-**The environmental nitrogen deposition in the Tibetan Plateau is about 8 kg N ha^-1^ yr^-1^, mainly in the form of NH_4_-N and NO_3_-N (Zhan et al. 2019). Therefore, three nitrogen deposition treatments were selected to simulate future atmospheric N deposition: N0 (0 kg ha^-1^ yr ^-1^), N16 (16 kg Nha^-1^ yr^-1^), and N32 (32 kg Nha^-1^ yr^-1^). Ammonium nitrate was used to simulate nitrogen deposition in the growing season. NH_4_NO_3_ was dissolved in water and evenly sprayed on the plot, and the same amount of water was also sprayed in the control. A randomized block design was used in this experiment. Four replicates were set in the plot, and 12 treatment levels were set in each plot, W0N0 ( no warming and nitrogen addition ), W0N16, W0N32, W1N0, W1N16, W1N32, W2N0, W2N16, W2N32, W3N0, W3N16, W3N32. A total of 48 samples were obtained.

**Question 8: Page 16:**

**How did you fit three 0.5 x 0.5 m quadrats into circle plot with diameter 0.95 m? Or I do not understand of the experimental design.**

**Response:	Thank you for your advice. This part of my description is not clear. Four replicates are set for each warming gradient, which means that there are four OTC per warming treatment. The area of each repeat sample is 0.5m × 0.5m. Modify as follows (L173-L175):**

**-**From 2023 to mid-August 2024, a sample plot survey was conducted at the peak of the vegetation growing season to obtain vegetation data. The sampling method was repeated four times, and the sampling area was 0.5 m × 0.5 m.

**Question 9: Page 17:**

**What does representative species for measurement of plant height mean? Do you mean five the most abundant species? How many individuals per species did you measure to get the plant height of one species?**

**Response:Thank you for your suggestion. Plant height determination means to determine the natural height of all species in the sample square, and 5 plants of each species are randomly selected to determine the height. The description of this part is not clear, and it has been modified. Modified as follows (L181-L184) :**

**-** Plant height determination refers to the determination of the natural height of all species in the quadrat. Five plants were randomly selected from each species to determine the height.

**Question 10: Page 18:**

**For trait measurement, you selected six from 15 recorded species. What does 15 recorded species mean? Fig. 2 shows that in total, there were 25 recorded species.**

**Why did you capitalize sedges, grasses and forbs?**

**You interchanged forbs and grasses. Poa pratensis is grass while the others are forbs.**

**Fig. 2:**

**The names of species should be in italic.**

**Response:Thank you for your advice. I misdescribed this part. The section has been reconfigured and redrawn. Modify as follows (L215-L221) :**

**-**From 2023 to August 2024, during a vegetation survey, six plant species were selected from the recorded 15 species for the measurement of functional traits. These species included Sedges (*Kobresia humilis*), Grasses (*Poa pratensis*), Forbs (*Potentilla anserina, Taraxacum lugubre, Gentiana futtereri, and Oxytropis ochrocephala*). The reason for determining these target species is that they are present in each plot, and these six plant species account for 80 % of the total plant species coverage (Fig.1).

Fig 1. Coverage of plant species in different plots

**Question 11: Page 20:**

**What does importance value mean? It was not described anywhere else. How did you calculate it?**

**Response:Thank you for your advice. Relative Importance value (IV) is the average of the sum of Relative Height (RH), Relative Coverage (RC) and Relative biomass (RB) in Wen Li (Plant species diversity and functional diversity relations in the degradation process of desert steppe in an In the study of arid area of northwest China et al., it was mentioned that "Importance value (IV) was used to show the community composition (gramineae, legumes and forbs) under different degradation gradients”**

**IV=RB +RH+ RC/3**

**RB, RH, and RC are the Relative biomass, relative height, and relative cover, respectively.By calculating the important values of different plant functional communities, we can understand the changes in the proportion of grasses, seperaceae and miscellaneous grasses in the whole plant community under temperature increase and nitrogen deposition, and can more intuitively understand the changes in plant community composition.**

**Question 12: Page 21:**

**Do you used Tukey test only for interaction of warming and nitrogen deposition or also for these two factors individually? It is not clear from the methodology. From results, it seams that you tested both interaction and individual factors, but the results are stated only in figures only for interactions.**

**Page 22:**

**Vegan package should be cited.**

**Response:Thanks for your suggestion, the statistical analysis section has been rewritten. Modify as follows (L255-L285) :**

**-**The data were evaluated for normality and mean square deviation before statistical analysis. The plant species diversity index, species richness index, species evenness index, the important value of Grasses, important value of Sedges, the important value of forbs, the weighted mean value of plant community functional traits, functional richness, functional evenness, functional divergence, functional dispersion, and Rao's secondary entropy were used for descriptive statistics. SPSS 25.0 software was used for linear mixed model analysis, with warming, nitrogen deposition, and year as fixed factors and block group as random factors. To understand the effects of warming, nitrogen deposition, and year changes on species diversity, functional traits, and functional diversity of plant communities. IBM SPSS Statistics (SPSS software version 25.0, Chicago, Illinois, USA) was used. Tukey's multiple comparison was performed, and the statistical test level was based on P < 0.05. The relationship between species diversity index and functional diversity index was fitted by Origin 2024. Species diversity and functional diversity were calculated using the ' vegan ' and ' FD ' packages in R version 4.2, respectively. Canoco 5.0 software was used to determine the effects of soil physical and chemical factors (soil water content, soil pH, soil bulk density, soil total nitrogen, soil total phosphorus, soil nitrate nitrogen, soil ammonium nitrogen, and soil organic carbon) on plant species diversity and functional diversity by redundancy analysis. Amos software ( IBM SPSS Amos 24.0 ) was used to construct a structural equation model to explore the influence path of each factor on soil species diversity and functional diversity, and the fitting of the model was judged by combining the performance indicators of the model, including the ratio of chi-square to degree of freedom ( less than 3 is good ), P value ( greater than 0.05 is good ), comparative fitting index (CFI, greater than 0.9 is good ), standardized root mean square residual ( SRMR, less than 0.1 is acceptable ) (Yang et al. 2020).In the final model, any non-significant relationship between variables is deleted. Except for the chi-square test of model fitting in SEM was set to *P* > 0.05, the significance level of all statistical tests was set to *P*<0.05. Except for structural equation modeling, all data analyses were performed in the R software environment (version 4.3.2). Before conducting structural equation model analysis, all response variables and predictors were standardized, and a z-score was used to represent the parameters to meet the hypothesis of the test used (Zuo et al. 2023).

**Question 13: Results
Describing the effect of warming and nitrogen deposition on CWM of traits, you did not present any statistical data (no results of statistical tests), only percentages and if it was or not significant. It should be somewhere, for example a table with the values for statistical test, df and p.
Fig. 3, 4, 5:
You did not state what the values for W, N and W*N mean. What was the test and what are df?
You did not describe what letters mean. It is the result of Tukey but for what? And why did you not compare also the differences between only warming treatment? You describe it in text, but in figures, you have results only for interactions.**

**Response: Thanks for your suggestion, the corresponding table has been added in the attachment, and the corresponding explanation has been made for the figure notes in the picture. Modified as follows:**

**-Attached table**：

Table S1 The results of the linear mixed effect model showed the effects of warming ( W ), nitrogen deposition ( N ), and their interaction ( W × N ) on plant species diversity in the first year ( 2023 ) and the second year ( 2024 ) of the experimental treatment. The p value ( < 0.05 ) was significant. df denotes degree of freedom

| Year | Treatment | df | Shannon-wiener’s diversity index | | Species richness index | | Species evenness index | | Important value of grasses | | Important value of sedges | | Important value of forb | |
| --- | --- | --- | --- | --- | --- | --- | --- | --- | --- | --- | --- | --- | --- | --- |
|  |  |  | F | P value | F | P value | F | P value | F | P value | F | P value | F | P value |
| 2023 | W | 3 | 12.03 | 0 | 3.41 | 0.13 | 3.67 | 0.10 | 48.31 | 0 | 3.30 | 0.03 | 2.97 | 0.04 |
|  | N | 2 | 1.08 | 0.23 | 0.99 | 0.40 | 3.47 | 0.11 | 31.31 | 0 | 1.20 | 0.52 | 0.78 | 0.48 |
|  | W×N | 6 | 0.51 | 0.54 | 0.35 | 0.96 | 1.33 | 0.29 | 5.90 | 0.04 | 2.37 | 0.04 | 1.32 | 0.27 |
| 2024 | W | 3 | 19.19 | 0 | 3.66 | 0.04 | 2.55 | 0.37 | 90.71 | 0 | 55.67 | 0 | 14.93 | 0 |
|  | N | 2 | 15.03 | 0 | 1.09 | 0.45 | 1.86 | 0.17 | 36.28 | 0 | 28.45 | 0 | 3.04 | 0.06 |
|  | W×N | 6 | 8.90 | 0.01 | 0.46 | 0.84 | 0.20 | 0.99 | 3.04 | 0.02 | 5.81 | 0.02 | 0.15 | 0.87 |
| Overall | Y | 1 | 1.04 | 0.67 | 0.09 | 0.77 | 1.28 | 0.50 | 1.21 | 0.57 | 1.21 | 0.61 | 1.03 | 0.68 |
|  | W | 3 | 20.92 | 0 | 7.68 | 0 | 1.99 | 0.11 | 58.54 | 0 | 28.01 | 0 | 48.82 | 0 |
|  | N | 2 | 19.23 | 0 | 3.11 | 0.07 | 1.16 | 0.60 | 34.36 | 0 | 17.11 | 0 | 2.95 | 0.06 |
|  | W×N | 6 | 5.57 | 0.03 | 0.09 | 0.74 | 2.01 | 0.10 | 4.35 | 0.01 | 2.30 | 0.17 | 0.50 | 0.47 |
|  | Y×N | 2 | 1.74 | 0.18 | 0.10 | 0.90 | 1.52 | 0.23 | 1.47 | 0.24 | 1.22 | 0.30 | 0.64 | 0.53 |
|  | Y×W | 3 | 5.28 | 0.08 | 0.58 | 0.97 | 0.59 | 0.62 | 2.44 | 0.44 | 4.55 | 0.07 | 0.94 | 0.57 |
|  | Y×W×N | 6 | 0.71 | 0.64 | 0.09 | 0.99 | 0.52 | 0.69 | 0.97 | 0.45 | 1.46 | 0.51 | 0.63 | 0.70 |

Table S2 The results of the linear mixed effect model showed the effects of warming ( W ), nitrogen deposition ( N ), and their interaction ( W × N ) on plant functional diversity in the first year ( 2023 ) and the second year ( 2024 ) of the experimental treatment. The p value ( < 0.05 ) was significant. df denotes degree of freedom

| Year | Treatment | df | Functional richness index | | Functional evenness index | | Functional divergence index | | Functional dispersion index | | Rao’s quadratic entropy index | |
| --- | --- | --- | --- | --- | --- | --- | --- | --- | --- | --- | --- | --- |
|  |  |  | F | P value | F | P value | F | P value | F | P value | F | P value |
| 2023 | W | 3 | 7.28 | 0 | 8.87 | 0 | 1.19 | 0.11 | 60.77 | 0 | 78.36 | 0 |
|  | N | 2 | 0.44 | 0.51 | 2.42 | 0.10 | 0.27 | 0.77 | 76.20 | 0 | 113.63 | 0 |
|  | W×N | 6 | 3.96 | 0.04 | 1.67 | 0.24 | 1.04 | 0.40 | 4.14 | 0.03 | 5.48 | 0.02 |
| 2024 | W | 3 | 9.03 | 0 | 7.88 | 0 | 1.64 | 0.14 | 49.09 | 0 | 44.73 | 0 |
|  | N | 2 | 5.72 | 0.03 | 1.86 | 0.22 | 1.81 | 0.25 | 24.87 | 0 | 22.11 | 0 |
|  | W×N | 6 | 6.71 | 0.004 | 4.11 | 0.03 | 1.98 | 0.15 | 2.93 | 0.04 | 2.63 | 0.04 |
| Overall | Y | 1 | 2.74 | 0.06 | 2.72 | 0.07 | 1.76 | 0.24 | 2.11 | 0.10 | 1.91 | 0.44 |
|  | W | 3 | 7.91 | 0 | 9.61 | 0 | 1.37 | 0.37 | 98.76 | 0 | 95.07 | 0 |
|  | N | 2 | 4.48 | 0.04 | 1.21 | 0.31 | 1.20 | 0.28 | 72.74 | 0 | 69.67 | 0 |
|  | W×N | 6 | 5.31 | 0.01 | 5.38 | 0.004 | 1.84 | 0.44 | 1.48 | 0.10 | 3.36 | 0.03 |
|  | Y×N | 2 | 1.62 | 0.15 | 1.48 | 0.24 | 1.78 | 0.44 | 1.46 | 0.06 | 2.53 | 0.40 |
|  | Y×W | 3 | 2.18 | 0.08 | 1.32 | 0.32 | 1.57 | 0.07 | 1.16 | 0.30 | 1.91 | 0.30 |
|  | Y×W×N | 6 | 1.92 | 0.13 | 2.23 | 0.07 | 1.13 | 0.36 | 2.07 | 0.07 | 2.07 | 0.06 |

Table S3 The results of the linear mixed effect model showed the effects of warming ( W ), nitrogen deposition ( N ), and their interaction ( W × N ) on the weighted average of plant community functional traits in the first year ( 2023 ) and the second year ( 2024 ) of the experimental treatment. The p value (< 0.05) was significant. df denotes degree of freedom

| Year | Treatment | dF | CWM_VH | | CWM_LA | | CWM_LL | | CWM_LW | | CWM_LDMC | | CWM_SLA | | CWM_ LPC | | CWM_LNC | | | CWM_LCC | |
| --- | --- | --- | --- | --- | --- | --- | --- | --- | --- | --- | --- | --- | --- | --- | --- | --- | --- | --- | --- | --- | --- |
|  |  |  | F | P | F | P | F | P | F | P | F | P | F | P | F | P | F | P | | F | P |
| 2023 | W | 3 | 21.22 | 0 | 18.89 | 0 | 12.71 | 0 | 2.91 | 0.05 | 23.11 | 0 | 3.95 | 0.01 | 0.75 | 0.41 | 100.13 | 0 | | 2.43 | 0.13 |
|  | N | 2 | 2.18 | 0.13 | 24.03 | 0 | 9.88 | 0 | 1.03 | 0.37 | 12.62 | 0 | 0.29 | 0.75 | 1.25 | 0.39 | 100.83 | | 0 | 0.06 | 0.58 |
|  | W×N | 6 | 1.43 | 0.23 | 1.45 | 0.22 | 0.31 | 0.93 | 1.82 | 0.12 | 4.36 | 0.002 | 2.75 | 0.02 | 1.30 | 0.44 | 7.55 | 0 | | 1.63 | 0.32 |
| 2024 | W | 3 | 39.74 | 0 | 30.53 | 0 | 29.93 | 0 | 2.56 | 0.07 | 16.72 | 0.01 | 13.64 | 0 | 1.94 | 0.13 | 82.45 | 0 | | 89.92 | 0 |
|  | N | 2 | 9.21 | 0.02 | 60.35 | 0 | 22.16 | 0 | 2.81 | 0.06 | 56.73 | 0 | 86.48 | 0 | 2.33 | 0.15 | 46.71 | 0 | | 53.28 | 0 |
|  | W×N | 6 | 12.52 | 0 | 8.62 | 0 | 17.91 | 0 | 1.25 | 0.33 | 5.56 | 0 | 81.40 | 0 | 1.48 | 0.44 | 96.30 | 0 | | 44.10 | 0 |
| Overall | Y | 1 | 2.62 | 0.27 | 2.42 | 0.12 | 1.21 | 0.45 | 0.60 | 0.51 | 0.52 | 0.47 | 1.80 | 0.25 | 1.42 | 0.31 | 1.48 | 0.28 | | 1.81 | 0.35 |
|  | W | 3 | 57.29 | 0 | 50.92 | 0 | 27.82 | 0 | 1.25 | 0.57 | 24.05 | 0 | 29.59 | 0 | 1.73 | 0.35 | 29.89 | 0 | | 11.54 | 0 |
|  | N | 2 | 5.14 | 0.03 | 25.89 | 0 | 27.94 | 0 | 1.33 | 0.41 | 19.60 | 0 | 23.58 | 0 | 2.18 | 0.44 | 37.52 | 0 | | 27.91 | 0 |
|  | Y×W | 6 | 1.72 | 0.30 | 1.03 | 0.47 | 1.03 | 0.48 | 1.63 | 0.29 | 1.64 | 0.29 | 1.44 | 0.41 | 1.87 | 0.28 | 1.40 | 0.29 | | 1.63 | 0.34 |
|  | Y×N | 2 | 1.27 | 0.47 | 1.69 | 0.35 | 1.29 | 0.41 | 1.34 | 0.39 | 1.01 | 0.28 | 1.87 | 0.36 | 1.67 | 0.24 | 1.27 | 0.37 | | 1.52 | 0.25 |
|  | W×N | 3 | 7.82 | 0 | 8.27 | 0 | 12.97 | 0 | 1.49 | 0.34 | 13.35 | 0.04 | 13.19 | 0.03 | 1.21 | 0.35 | 33.21 | 0 | | 21.62 | 0 |
|  | Y×W×N | 6 | 1.47 | 0.36 | 1.45 | 0.28 | 1.18 | 0.16 | 1.61 | 0.36 | 1.54 | 0.28 | 1.03 | 0.34 | 1.37 | 0.29 | 1.23 | 0.19 | | 1.37 | 0.43 |

Note: CWM_VH, CWM_LA, CWM_LL, CWM_LW, CWM_LDMC, CWM_SLA, CWM_ LPC, CWM_LNC,CWM_LCC are expressed as weighted mean of plant height, weighted mean of leaf area, weighted mean of leaf length, weighted mean of leaf width, weighted mean of leaf weight, respectively. Weighted mean of specific leaf area, weighted mean of leaf phosphorus, weighted mean of leaf nitrogen, weighted mean of leaf carbon

**-Result analysis section(L287-L321)：**

Effects of warming and nitrogen deposition on plant species diversity in alpine meadow

In 2023, compared with W0, the Shannon-Wiener diversity index increased significantly by 7.74 % under W1 treatment, and decreased significantly by 2.61 % under W3 treatment. The important value of grasses was significantly increased by 37.38 % and 40.83 % under W2 and W3 treatments, respectively. The importance value of forbs decreased significantly by 41.20 % under the W3 treatment. The importance value of sedges decreased significantly by 24.68 %, 37.74 %, and 42.34 % under W1, W2, and W3 treatments, respectively. Compared with N0, the importance value of grasses increased significantly by 18.52 % and 69.72 % under N16 and N32 treatments, respectively. The interaction of warming and nitrogen deposition will significantly affect the importance value of grasses, sedges, and forbs. The importance value of grasses reached the maximum under the W3N32 treatment, the importance value of sedges reached the minimum under the W3N32 treatment, and the importance value of forbs reached the minimum under the W2N32 treatment.

In 2024, Compared with W0, the Shannon-Wiener diversity index increased significantly by 1.76 % and 3.53 % under W1 and W2 treatments and decreased significantly by 3.02 % under W3 treatment. The species richness index increased significantly by 27.5 % under the W1 treatment. The important value of grasses was significantly increased by 55.42 %, 111.78 %, and 153.93 % under W1, W2, and W3 treatments, respectively. The important value of forbs was significantly reduced by 34.29 % and 43.18 % under W2 and W3 treatments. The importance value of sedges decreased significantly by 19.54 %, 33.37 %, and 53.64 % under W1, W2, and W3 treatments, respectively. Compared with N0, the Shannon-Wiener diversity index decreased significantly by 7.49 % under the N32 treatment. The importance value of grasses increased significantly under N32 treatment. The importance value of sedges decreased significantly by 62.95 % under N32 treatment. The interaction of warming and nitrogen deposition will significantly affect the Shannon-Wiener diversity index, the importance value of grasses, and the importance value of sedges. The Shannon-Wiener diversity index and the importance value of forbs reached the minimum value under the W3N32 treatment, and the importance value of grasses reached the maximum value under the W3N32 treatment.

The results of the mixed linear model showed that interannual variation had no significant effect on plant species diversity index and plant functional groups(Table S1,Fig.2).

Fig. 2 Effects of warming and nitrogen deposition on plant species diversity

Different lowercase letters indicated that there were significant differences in different nitrogen deposition treatments under the same warming level, and different uppercase letters indicated that there were significant differences in different warming treatments under the same nitrogen deposition level (^ns^*P*>0.05; ^*^*P* < 0.05; *^**^ P* < 0.01; ^* * *^ *P* < 0.001). The same below

**Question 14: Page 29:
There is no df for Monte Carlo permutation test (“(F = 2.2, P= 0.016)”).
Fig. 6:
c) Is it necessary to show so many decimals for in legend for Mantel´s r?
Response: Thank you for your advice. By rearranging the statistical analysis part, the corresponding result analysis part has been modified as follows (L413-L473):**

**-**Response mechanism of plant species diversity and functional diversity to warming and nitrogen deposition

The relationship between plant species diversity index, functional diversity index, plant community functional traits, and soil physical and chemical properties was analyzed by redundancy analysis. Monte Carlo results showed that STN, CWM _ LNC, grasses, SC / N, SAN, and CWM _ SLA were the key factors in determining the plant species diversity index. Among them, the Shannon-Wiener diversity index and species evenness index of plants were negatively correlated with STN, SAN, CWM _ LNC, and CWM _ SLA, and positively correlated with Grasses and SC / N ( Figure 5-a, Table 2 ). The plant functional diversity index was mainly affected by grass, SC / N, CWM _ LDMC, SAN, CWM _ LA, SWC, SOC, SC / P, and SN / P ( Figure 5-b, Table 2 ).

The direct and indirect effects of plant and soil characteristics on plant species diversity and plant functional diversity under warming and nitrogen deposition were quantified by structural equation modeling (Fig.6 ). The results showed that warming and nitrogen deposition had significant negative effects on plant species diversity (path coefficients were -0.73 and -0.84, respectively). Warming and nitrogen deposition had a significant positive correlation with soil physical and chemical properties (path coefficients were 0.76 and 0.21, respectively), had a significant positive correlation with plant traits (path coefficients were 0.46 and 0.64, respectively), and negatively affected plant species diversity index through plant and soil characteristics (path coefficients were-0.39 and-0.26, respectively). Warming and nitrogen deposition had significant negative effects on plant functional diversity (path coefficients were-0.40 and-0.33, respectively), and negatively affected plant functional diversity index through plant and soil characteristics (path coefficients were 0.69 and 0.25, respectively ). The results also showed that plant functional diversity in alpine meadows was positively affected by plant species diversity under warming and nitrogen deposition (path coefficient was 0.56 ).In general, with the increase of warming and nitrogen deposition, the plant species diversity index and plant functional diversity index showed a decreasing trend. Soil factors (soil total nitrogen and soil carbon-nitrogen ratio ) and plant factors (Grasses importance value, leaf nitrogen weighted mean, specific leaf area-weighted mean, leaf area-weighted mean, and leaf weight weighted mean ) will have direct and indirect effects on plant species diversity and plant functional diversity through warming and nitrogen deposition.

Fig. 5 Redundancy analysis of plant species diversity, plant functional diversity, plant functional traits, and soil physical and chemical properties under warming and nitrogen deposition

STN, soil total nitrogen; cWM _ LNC, leaf nitrogen weighted mean; grasses, Grasses important value; SC / N, soil C / N ratio; cWM _ LW, leaf width weighted mean; sAN, soil ammonium nitrogen; sWC, soil moisture content; forb, the important value of forbs, CWM _ VH, the weighted mean of plant height; cWM _ SLA, weighted mean of specific leaf area; sT, soil temperature; sBD, soil bulk density; sTC, soil total carbon; sAP, soil available phosphorus; sTP, soil total phosphorus; SC / P, soil carbon and phosphorus ratio; sOC, soil organic carbon; sedge, sedge important value; cWM _ LCC, leaf carbon weighted mean; cWM _ LL, leaf length weighted mean; cWM _ LA, weighted mean of leaf area; cWM _ LDMC, leaf weight weighted mean; sNN, soil nitrate nitrogen; cWM _ LPC, leaf phosphorus weighted mean; pH, soil pH, the same below. Red and black arrows represent plant functional traits, soil physical and chemical properties ( explanatory variables ), and plant species diversity index and plant functional diversity ( response variables ), respectively. The cosine value of the angle between the two lines indicates the correlation between the two. The length of the explanatory variable line indicates the degree of interpretation of the response variable. The longer the line segment, the higher the degree of interpretation.

Table 2 Redundancy analysis between plant diversity index, plant functional diversity index, soil physicochemical properties, and plant characteristics

| Factor | variable | Explain（%） | Contribution（%） | Pseudo-F | P |
| --- | --- | --- | --- | --- | --- |
| plant  species  diversity  index | STN | 6.8 | 16.8 | 6.8 | 0.008 |
|  | CWM_LNC | 4.2 | 10.3 | 4.3 | 0.04 |
|  | grasses | 17.7 | 8.0 | 7.2 | 0.001 |
|  | SC/N | 3.7 | 9.2 | 4.3 | 0.044 |
|  | CWM_LW | 2.4 | 6.0 | 2.9 | 0.053 |
|  | SAN | 3.5 | 8.7 | 4.3 | 0.042 |
|  | SWC | 1.6 | 4.0 | 2.0 | 0.064 |
|  | forb | 1.3 | 3.1 | 1.6 | 0.231 |
|  | CWM_VH | 1.3 | 3.2 | 1.6 | 0.214 |
|  | CWM_SLA | 2.7 | 6.7 | 3.5 | 0.047 |
|  | ST | 1.2 | 3.0 | 1.6 | 0.186 |
|  | SBD | 0.6 | 1.5 | 0.8 | 0.404 |
|  | STC | 0.6 | 1.4 | 0.7 | 0.406 |
|  | SAP | 0.1 | 0.4 | 0.2 | 0.67 |
|  | STEP | 0.3 | 0.8 | 0.4 | 0.54 |
|  | SC/P | 0.8 | 2.1 | 1.1 | 0.332 |
|  | SOC | 0.2 | 0.4 | 0.2 | 0.63 |
|  | sedge | 0.1 | 0.3 | 0.1 | 0.716 |
|  | CWM_LCC | <0.1 | 0.1 | <0.1 | 0.885 |
|  | CWM_LL | 0.1 | 0.3 | 0.2 | 0.71 |
|  | CWM_LA | 0.2 | 0.5 | 0.2 | 0.64 |
|  | CWM_LDMC | <0.1 | 0.2 | 0.1 | 0.742 |
|  | SNN | <0.1 | <0.1 | <0.1 | 0.882 |
|  | CWM_LPC | <0.1 | <0.1 | <0.1 | 0.915 |
|  | PH | <0.1 | <0.1 | <0.1 | 0.934 |
| Plant  functional  diversity  index | grass | 54.2 | 66.0 | 110 | 0.002 |
|  | SC/N | 10.5 | 12.7 | 27.3 | 0.009 |
|  | CWM_LDMC | 3.6 | 4.4 | 10.3 | 0.012 |
|  | SAN | 1.7 | 2.0 | 5.0 | 0.018 |
|  | CWM_LA | 1.5 | 1.8 | 4.6 | 0.018 |
|  | SWC | 1.4 | 1.7 | 4.5 | 0.017 |
|  | SOC | 1.0 | 1.3 | 3.5 | 0.042 |
|  | SC/P | 1.7 | 2.1 | 5.9 | 0.010 |
|  | SN/P | 1.0 | 1.2 | 3.6 | 0.044 |
|  | CWM_LCC | 0.8 | 1.0 | 2.9 | 0.061 |
|  | CWM_LW | 0.7 | 0.9 | 2.7 | 0.078 |
|  | ST | 0.5 | 0.6 | 1.9 | 0.214 |
|  | STC | 0.4 | 0.4 | 1.4 | 0.278 |
|  | CWM_LL | 0.4 | 0.5 | 1.4 | 0.228 |
|  | PH | 0.3 | 0.4 | 1.2 | 0.284 |
|  | CWM_LPC | 0.3 | 0.4 | 1.1 | 0.324 |
|  | CWM_LNC | 0.3 | 0.3 | 1.0 | 0.327 |
|  | forb | 0.3 | 0.4 | 1.2 | 0.288 |
|  | CWM_VH | 0.2 | 0.2 | 0.7 | 0.482 |
|  | CWM_SLA | 0.1 | 0.2 | 0.5 | 0.534 |
|  | SNN | 0.1 | 0.2 | 0.5 | 0.538 |
|  | SAP | 0.2 | 0.2 | 0.6 | 0.498 |
|  | STN | <0.1 | 0.1 | 0.4 | 0.571 |
|  | SBD | <0.1 | <0.1 | 0.2 | 0.776 |
|  | sedges | <0.1 | <0.1 | 0.2 | 0.764 |

Fig.6 The structural equation model showed the direct and indirect effects of plant factors and soil factors on plant species diversity and plant functional diversity

( a ) Structural equation model ; ( b ) The total effect, direct effect, and indirect effect of plant and soil factors on plant species diversity. ( c ) The total effect, direct effect and indirect effect of plant and soil factors on plant functional diversity. Red and black arrows indicated a negative and positive relationship ( P < 0.05 ). The width of the arrow is proportional to the strength of the path coefficient, and the adjacent numbers on the arrow represent the standardized path coefficient. R2 represents the proportion of variance explained by the model. * * *, *P* < 0.001; * *, *P* < 0.01 ; *, *P* < 0.05.

**Question 15: Page 32: I am confused about the community height and height measured as trait. It is not appropriately differed and from results, I had the impression that you did not use the community height. But now, it is in discussion, but I did not find nothing about community height in the results.**

**Response: Thank you for your advice. Community height was determined by "quantifying the average weighted traits of plant communities in each subplot:**

**This formula is calculated. All the weighted average values of plant community functional traits in this paper were calculated using this formula.
Question 16: Page 37:
You are stated that the water availability is a critical limiting factor for plant growth, but you are not discussing it with your results.**

**Response:Thank you for your advice. Re-organize the discussion by reading the relevant literature. Modify as follows (L490-L500) :**

**-**First of all, temperature is a key limiting factor for the growth of alpine plants, but excessive temperature may affect the growth of temperature-sensitive plant species(Wang et al. 2022a). Secondly, water availability is another key limiting factor for plant growth. Drying may lead to stomatal closure and photorespiration, thus affecting the growth of plant species(Grant et al. 2017, Han et al. 2023). Combined with the above, the difference in temperature and humidity in the process of warming will affect the plant community structure and species composition, but different plant species have different sensitivity to temperature. As a result, some plant species with poor resistance to temperature and stress exceeded the threshold of the adaptation range, resulting in a decrease in species dominance and loss of species, which eventually led to changes in community structure (Xu et al. 2020).

**Question 17: Supporting information
In table with data, there are some names of variables only in Chinese. It should be in English.**

**Response:Thank you for your advice. The variable names in the supplementary information have been translated into English to improve readability.**

**We believe that these revisions have significantly enhanced our manuscript and thank the reviewers again for their valuable suggestions.**

**Reviewer3:**

**Question 1: This article assesses the impact of warming and nitrogen deposition on plant traits, soil properties and species community assembly in an experiment carried out on alpine grasslands of the Qinghai-Tibet Plateau. I enjoyed reading this manuscript, the text is well written and the experiment represents a large amount of effort. The discussion section could be improved to link it better to the research questions. The graphs could also be clearer, particularly with reference to the results of the Tukey test. I have provided more details below and made some other suggestions for further improvements.**

**Materials and methods**

**In the section ‘Plant Community Structure Survey and Analysis’ the meanings of the letters C and B in the first formula are missing.**

**Response:Thank you for your advice. After reading relevant literature, relevant parts have been modified. Modify as follows (L200-L206) :**

**-**1) Species diversity of plant community

Species diversity within the community was evaluated using alpha diversity indices, including 1) Species richness index (R); 2) Shannon-Weiner index (H); and 3) Pielou's evenness index (J). The formulas are as follows (Zhang et al. 2022b) ：

Importance value (IV) : is the mean value of the sum of relative height ( RH ), relative coverage ( RC ), and relative biomass ( RB ).

**Question 2: Some of the methods mentioned do not have a reference associated with them e.g. the molybdenum antimony anti-colorimetric method, hydrazine sulfate method and the indophenol blue method. Please add appropriate references for these techniques.**

**Response:Thank you for your advice. After reading the relevant literature, the corresponding references have been added. Modified as follows (L186-L198):**

**-**Determination of soil physical and chemical properties

In August 2023-2024, samples were collected during the plant growth season, and a 0.5 × 0.5 m sample box was randomly placed in each plot. Five soil cores with a diameter of 3 cm and a depth of 30 cm were drilled in each sample. After mixing them, the roots and stones of the plants were removed with a 2 mm mesh sieve for the determination of soil physical and chemical properties. Soil water content ( SWC ) was measured by oven-drying method ( 105 °C, 24 h ). Soil pH ( pH ) was measured by potentiometric method ( soil-water ratio was 1 : 2.5 ). Soil bulk density ( SBD ) was measured by ring knife method. Soil total phosphorus ( STP ) was determined by Mo-Sb colorimetric method. Soil ammonium nitrogen ( SAN ) was determined by potassium chloride extraction-indophenol blue colorimetric method (Zhang et al. 2021) ; soil nitrate nitrogen ( SNN ) was determined by dual-wavelength ultraviolet spectrophotometer (Wang et al. 2022b).

**Question 3: Figure 3 – the figure legend does not match all the graphs presented. CWM_LW (leaf width), CWM_LPC (leaf phosphorus concentration) and CWM_LCC (leaf carbon concentration) are listed in the legend but are not part of the graphs. Instead CWM_LC/N and CWM_LC/P are in the graphs but not in the legend. Please correct.**

**Response:Thank you for your advice. The legend in Figure 3 has been modified and kept consistent throughout the text to increase readability. Modified as follows (L329-L388):**

**-**Effects of warming and nitrogen deposition on plant functional diversity in alpine meadow

In 2023, Compared with W0, the plant functional richness index was significantly reduced by 58.69 % in the W3 treatment; the plant functional evenness index decreased significantly by 27.94 %, 19.15 %, and 9.91 % under W1, W2, and W3 treatments. The plant functional dispersion index decreased significantly by 54.39 %, 47.22 %, and 32.64 % under W1, W2 and W3 treatments. The RaoQ secondary entropy index was significantly reduced by 57.62 %, 67.83 %, and 74.21 % under W2 and W3 treatments. The plant height-weighted mean, leaf area-weighted mean, leaf length-weighted mean, leaf weight-weighted mean, and leaf nitrogen-weighted mean were significantly increased by 3.18 %, 61.26 %, 85.82 %, 152.78 %, and 13.64 % under W3 treatment, respectively. The weighted mean of specific leaf area was significantly reduced by 54.83 % under W3 treatment. Compared with N0, the plant functional dispersion index, and RaoQ secondary entropy index were significantly reduced by 10.11 % and 9.84 % under the N32 treatment. The leaf area-weighted mean, leaf length-weighted mean, leaf weight-weighted mean, and leaf nitrogen-weighted mean were significantly increased by 125.99 %, 131.14 %, 81.29 %, 62.52 %, 97.72 %, and 9.91 % under N32 treatment, respectively. The interaction of warming and nitrogen deposition also significantly affected the plant functional richness index, plant functional dispersion index, RaoQ secondary entropy index, leaf weight weighted mean, specific leaf area-weighted mean, and leaf nitrogen weighted mean. The plant functional richness index reached the minimum value under the W316 treatment, and the plant functional dispersion index and RaoQ secondary entropy index reached the maximum value under the W3N32 treatment. The weighted mean of leaf weight and leaf nitrogen reached the minimum under the W3N32 treatment.

In 2024, Compared with W0, the plant functional richness index was significantly reduced by 44.73 %, 51.13 %, and 52.49 % under W1, W2, and W3 treatments; the plant functional evenness index was significantly reduced by 14.01 % and 16.16 % under W2 and W3 treatments. The plant functional dispersion index was significantly reduced by 20.95 %, 41.44 %, and 43.55 % under W1, W2, and W3 treatments. The quadratic entropy index of RaoQ significantly increased by 19.36 %, 47.23 %, and 51.22 % under the W1, W2, and W3 treatments. The weighted mean of plant height increased significantly by 182.59 % and 71.24 % under W2 and W3 treatments. The weighted mean of leaf area increased significantly by 73.84 % under W3 treatment. The weighted mean of leaf length increased significantly by 67.55 %, 151.78 %, and 230.31 % in W1, W2, and W3 treatments, respectively. The weighted mean of leaf weight increased significantly by 144.27 % and 209.28 % under W2 and W3 treatments. The weighted mean of specific leaf area was significantly reduced by 13.38 % and 2.99 % under W2 and W3 treatments. The weighted mean of leaf nitrogen was significantly reduced by 10.36 % and 16.66 % under W2 and W3 treatments. The weighted mean of leaf carbon increased significantly by 26.67 % and 28.03 % under W1, W2 and W3 treatments. Compared with N0, the plant functional richness index was significantly reduced by 57.29 % under N32 treatment. The weighted mean of plant height, weighted mean of leaf area, weighted mean of leaf length, weighted mean of leaf weight, weighted mean of specific leaf area, weighted mean of leaf nitrogen, and weighted mean of leaf carbon increased significantly by 63.53 %, 13.32 %, 93.65 %, 159.04 %, 20.76 %, 4.71 % and 26.07 % respectively under N32 treatment. The plant functional dispersion index and RaoQ secondary entropy index decreased by 10.27 % and 11.45 % respectively under N32 treatment. Under the W3N32 treatment, the plant function richness index, plant function dispersion index, and RaoQ secondary entropy index reached the minimum value. Under the W2N32 treatment, the plant function uniformity reached the minimum value. The weighted mean of specific leaf area and leaf carbon reached the minimum value under W2N16 treatment. The plant height-weighted mean, leaf area-weighted mean, leaf length-weighted mean, leaf weight-weighted mean, and leaf nitrogen-weighted mean reached the maximum under W3N32 treatment. The results of the mixed linear model showed that interannual variation had no significant effect on plant functional diversity and mean functional traits (Table S2,Table S3, Fig.3).

Fig. 3 Effects of temperature increase and nitrogen deposition on plant functional diversity

**Question 4:Figures 3, 4 and 5 – it is not clear what the Tukey test letters refer to. Please add a note in the figure legend clarifying the difference between the upper and lower case letters.**

**Response:Thank you for your advice. The meanings represented by the letters in Figures 3, 4, and 5 have been added. The specific changes are as follows (L287-328) :**

**-**Effects of warming and nitrogen deposition on plant species diversity in alpine meadow

In 2023, compared with W0, the Shannon-Wiener diversity index increased significantly by 7.74 % under W1 treatment, and decreased significantly by 2.61 % under W3 treatment. The important value of grasses was significantly increased by 37.38 % and 40.83 % under W2 and W3 treatments, respectively. The importance value of forbs decreased significantly by 41.20 % under the W3 treatment. The importance value of sedges decreased significantly by 24.68 %, 37.74 %, and 42.34 % under W1, W2, and W3 treatments, respectively. Compared with N0, the importance value of grasses increased significantly by 18.52 % and 69.72 % under N16 and N32 treatments, respectively. The interaction of warming and nitrogen deposition will significantly affect the importance value of grasses, sedges, and forbs. The importance value of grasses reached the maximum under the W3N32 treatment, the importance value of sedges reached the minimum under the W3N32 treatment, and the importance value of forbs reached the minimum under the W2N32 treatment.

In 2024, Compared with W0, the Shannon-Wiener diversity index increased significantly by 1.76 % and 3.53 % under W1 and W2 treatments and decreased significantly by 3.02 % under W3 treatment. The species richness index increased significantly by 27.5 % under the W1 treatment. The important value of grasses was significantly increased by 55.42 %, 111.78 %, and 153.93 % under W1, W2, and W3 treatments, respectively. The important value of forbs was significantly reduced by 34.29 % and 43.18 % under W2 and W3 treatments. The importance value of sedges decreased significantly by 19.54 %, 33.37 %, and 53.64 % under W1, W2, and W3 treatments, respectively. Compared with N0, the Shannon-Wiener diversity index decreased significantly by 7.49 % under the N32 treatment. The importance value of grasses increased significantly under N32 treatment. The importance value of sedges decreased significantly by 62.95 % under N32 treatment. The interaction of warming and nitrogen deposition will significantly affect the Shannon-Wiener diversity index, the importance value of grasses, and the importance value of sedges. The Shannon-Wiener diversity index and the importance value of forbs reached the minimum value under the W3N32 treatment, and the importance value of grasses reached the maximum value under the W3N32 treatment.

The results of the mixed linear model showed that interannual variation had no significant effect on plant species diversity index and plant functional groups(Table S1,Fig.2).

Fig. 2 Effects of warming and nitrogen deposition on plant species diversity

Different lowercase letters indicated that there were significant differences in different nitrogen deposition treatments under the same warming level, and different uppercase letters indicated that there were significant differences in different warming treatments under the same nitrogen deposition level (^ns^P>0.05; *P < 0.05; ** P < 0.01; * * * P < 0.001). The same below

**Question 5:Under the section “Effects of Warming and Nitrogen Deposition on Plant Community Species Diversity” the sentence “Nitrogen deposition also significantly affects the importance values of forbs and sedges” is repeated, please delete.**

**Response:Thank you for your advice. Duplicate parts have been deleted.**

**Question 6:Discussion
At the beginning of the discussion it would be beneficial to have a short summary paragraph of the overall findings before going into the details. The discussion is presented in such a way that seems to highlight previous research findings before talking about your results. I would re-frame this to first emphasise the findings from your study and then explain the results in the context of previously published research.**

**Question 7:At the end of the first paragraph of the discussion you mention “intraspecific trait responses to temperature changes vary among different species.”. Did you check for intraspecific variation in traits? It could be that there is a large trait variation within species or that one particular species is driving the changes observed.**

**Response:Thank you for your advice. The discussion part is not comprehensive due to the insufficient reading of relevant literature in the early stage. Reframe the discussion section. The overall approach is to highlight the findings first and then interpret them in the context of previously published research. Modified as follows (L475-L708) :**

**-**Discussion

Effects of warming and nitrogen deposition on plant community species diversity

Plant species diversity is an important factor affecting the complexity and stability of community function (Cardinale et al. 2012). The results of this study showed that the Shannon-Weiner index increased significantly and then decreased with the increase of the warming gradient, and the plant species richness index increased significantly under the W1 treatment. Studies have shown that warming has a significant negative impact on global grassland species richness, but has no effect on plant Shannon-Wiener index and species evenness(Yue et al. 2020). In the 4-year warming experiment on the alpine meadow, it was found that warming had no significant effect on species richness, Shannon diversity, and Shannon evenness (Hu et al. 2021). Studies in the Loess Plateau have shown that low-level warming increases plant community species diversity, but high-level warming reduces plant species diversity(Yang et al. 2018). This shows that compared with low-level warming, high-level warming has a greater impact on plant community species diversity. First of all, temperature is a key limiting factor for the growth of alpine plants, but excessive temperature may affect the growth of temperature-sensitive plant species(Wang et al. 2022a). Secondly, water availability is another key limiting factor for plant growth. Drying may lead to stomatal closure and photorespiration, thus affecting the growth of plant species(Grant et al. 2017, Han et al. 2023). Combined with the above, the difference in temperature and humidity in the process of warming will affect the plant community structure and species composition, but different plant species have different sensitivity to temperature. As a result, some plant species with poor resistance to temperature and stress exceeded the threshold of the adaptation range, resulting in a decrease in species dominance and loss of species, which eventually led to changes in community structure(Xu et al. 2020). The results of this study also showed that warming significantly increased the importance value of grasses and decreased the importance value of sedges and forbs. In the study of alpine meadow communities, it was also shown that warming increased the importance value of grasses and decreased the importance value of sedges(Peng et al. 2017). Studies have also shown that plant communities will change from weed-based to grass-based under warming conditions, while sedge and legume species remain relatively unchanged in response to warming(Ganjurjav et al. 2016, Liu et al. 2018). This is mainly because the response of different plant species to climate warming is mainly caused by the differences in biological characteristics and resource utilization of functional group plants and the response to environmental changes(Ma et al. 2022).

Many scholars believe that increased nitrogen deposition is one of the important factors affecting the species composition and diversity of plant communities (Zhou et al. 2018, Han et al. 2019). This study found that in 2024 when the nitrogen addition amount was 32kg.Nha-1year-1, the plant Shannon-Weiner index would be significantly reduced. Studies have shown that under a low nitrogen addition rate ( ≤ 10 g m-2 yr-1 ), the negative effect of light competition on species richness is more significant, while under a high nitrogen addition rate ( > 10 g m-2 yr-1 ), the negative effect of soil ion toxicity is more significant, indicating that with the increase of soil nitrogen availability caused by nitrogen addition, the transition from light competition to ion toxicity occurs(Namuhan et al. 2024). The results of this study showed that nitrogen deposition significantly increased the importance value of Grasses, but decreased the importance value of Sedges. Studies have shown that nitrogen deposition will enable dominant species to obtain more nutrients for rapid growth, thereby reducing the community species diversity index by reducing rare species(Zhang et al. 2022a). The fierce competition within the plant community has prompted nitrogen-preferred plants to obtain more light and water resources. For example, higher species shade shorter species, and shorter plants are excluded from the community due to light restrictions, eventually leading to a decline in species diversity(Zhang et al. 2015). At the same time, the increase of soil available nitrogen may accelerate the growth of resource-acquired species, and the return on nutrient investment is faster, while inhibiting the growth of resource-saving species, resulting in a decrease in plant diversity(Suding et al. 2005).

The results of this study also showed that the interaction of warming and nitrogen deposition would significantly affect the Shannon-Wiener diversity index of plants, the importance value of forbs, and the importance value of grasses. Previous studies have also shown that plant species diversity is affected by the interaction of warming and nitrogen deposition (Xu et al. 2018). The interaction between nitrogen deposition and warming is mainly attributed to the complex and indirect effects of warming. On the one hand, due to the different sensitivity of species to temperature, the trait changes caused by warming are usually species-specific(Elmendorf et al. 2012). On the other hand, warming can affect soil nitrogen availability, which in turn affects plant growth(Lu et al. 2013).

Effects of warming and nitrogen deposition on plant functional diversity

Plant functional traits are of great significance for exploring the relationship between plants and the environment, and provide a basis for predicting the response of ecosystems in the context of global change(Li et al. 2015). The results of this study showed that warming would increase the plant height-weighted mean, leaf area-weighted mean, leaf length-weighted mean, leaf-weight-weighted mean, and leaf nitrogen-weighted mean of the plant community. Climate warming will shift the mean of weighted traits of plant communities in a more accessible direction, including higher plants, larger leaves, and higher leaf nutrient concentrations(Salmon et al. 2016, Baruah et al. 2017). This study area belongs to the permafrost region. Low temperature will hinder plant growth and produce conservative traits(Wright et al. 2017), while warming will reduce the temperature limit of plant species in alpine meadows and promote the change of plant leaf morphology ( area, size, and width ) (Wei et al. 2023).

The results of this study also showed that the weighted mean of leaf area, the weighted mean of leaf length, the weighted mean of leaf weight, and the weighted mean of leaf nitrogen increased significantly when the nitrogen addition amount was 32kg.Nha-1year-1. Studies on the Qinghai-Tibet Plateau have also shown that nitrogen deposition can directly affect plant functional traits related to light acquisition or nitrogen availability ( such as plant height, leaf area, plant carbon, nitrogen, and phosphorus content, etc. )(Li et al. 2022). Nitrogen addition can increase the concentration of soil inorganic nitrogen, thereby increasing the reabsorption rate of plant nitrogen(Cao et al. 2021), which in turn increases the nitrogen content of plant leaves. To maintain the stoichiometric balance of plants, the demand for carbon and phosphorus increases with the increase in leaf nitrogen concentration (Zhang et al. 2017). At the same time, the plasticity of plant leaf functional traits enables plants to adapt to increased nitrogen deposition in the environment(Sun et al. 2022). Previous studies have found that the increase of nitrogen deposition increases the nitrogen concentration of plant green and senescent leaf tissues, and also changes the stoichiometric ratio of plant leaves (Cleveland et al. 2011). This study found that the interaction of warming and nitrogen deposition could significantly affect the functional traits of plant communities. Warming and nitrogen deposition have additive effects on plant community functional traits(Nogueira et al. 2018). This is mainly due to the different sensitivity of plant species to temperature, and the trait changes caused by warming are usually species-specific(Elmendorf et al. 2012). At the same time, warming will increase the availability of soil nitrogen, thereby enhancing the effect of nitrogen enrichment, which indicates that the effects of nitrogen enrichment and warming on the functional traits of alpine meadows are superimposed(Xu et al. 2018).

Compared with the weighted mean of community traits, the functional diversity of plant communities indicates that there are differences in the strategies of plant acquisition and utilization of resources(Diaz et al. 2007). The results of this study showed that warming significantly reduced the plant functional richness index. Some studies have found that functional diversity is higher in stressful environments, while in resource-limited habitats, restricted similarity may tend to select species with more diverse resource utilization strategies (Shovon et al. 2020).In contrast, other studies have suggested that under malnourished conditions, functional diversity is low, because strong environmental filtering allows only certain species to persist, which may lead to a convergence of resource utilization strategies for co-existing species in the community(Símová et al. 2015). This indicates that due to the need for plant species to adapt to external climate change, the functional traits of plant species are homogenized, which significantly reduces the niche of plant functional traits in the spatial range (Shipley et al. 2016). The RaoQ index and FDis index can be used to measure the degree of niche differentiation and resource competition among plants in the community(Li et al. 2024). The higher the index value, the stronger the niche complementarity between species, the weaker the competition, and the higher the resource utilization efficiency (Dong et al. 2017). The results of this study showed that nitrogen deposition significantly reduced the plant functional richness index, RaoQ secondary entropy index, and plant functional dispersion index. Biological processes such as competition can produce trait differentiation by excluding similar species ( i.e., promoting niche differences ) (Xu et al. 2018). This means that the dominant plants tend to eliminate dwarf plants with similar traits in the vicinity. Nitrogen addition may lead to the emergence of new environmental conditions conducive to the selection of new traits while placing common species at a disadvantage and accelerating competitive exclusion (Clark et al. 2007, DeMalach and Kadmon 2017).

The relationship between plant species diversity and plant functional diversity under warming and nitrogen deposition

Plant species diversity and functional diversity are important foundations of ecosystem functions and important indicators for evaluating ecosystem stability(Petchey and Gaston 2006). The results of this study show that with the increase of warming gradient, the correlation between plant functional differentiation index, plant functional dispersion index, RaoQ secondary entropy index, and species richness index is gradually weakened. Studies have also shown that plant species richness is closely related to functional richness(Naeem 2002). Because when the temperature is not increased, the plant community has the highest species richness and the largest number of species. The wider the distribution of functional traits in the plant community, the greater the functional niche occupied by the species (Biswas and Mallik 2011), and the correlation between the two is the strongest. However, with the increase in temperature, the number of species gradually decreased and the functional traits of species showed homogenization, which significantly reduced the spatial range of functional traits(Gravel et al. 2011), and the correlation was weakened. The results of this study also showed that with the increase of warming gradient and nitrogen deposition, the correlation coefficient between plant Shannon-Wiener index and plant functional richness index, plant functional dispersion index, and RaoQ secondary entropy index decreased gradually. Studies have shown that the relationship between plant species diversity and plant species functional diversity is different under different external environmental conditions(Mayfield et al. 2005). With the increase of climate warming and nitrogen deposition, species that adapt to changes in the external environment continue to grow, and these species have similar characteristics and show a high degree of redundancy in function. Studies have shown that grazing leads to the selection of palatable species, resulting in increased functional redundancy(Pakeman 2004). Functionally redundant species play the role of ' ecological insurance ' and maintain the stability of the ecosystem(Sasaki et al. 2009).In conclusion, as an important part of biodiversity, plant diversity plays an important role in maintaining the stability of grassland ecosystems. Phylogenetic diversity can reflect the ecological process of plant community composition and plays an important role in revealing the maintenance mechanism of biodiversity(Sun et al. 2021). Therefore, exploring the effects of warming and nitrogen deposition on plant phylogenetic diversity in future studies can better understand the corresponding mechanisms of plant diversity on climate change in grassland ecosystems.

Regulatory mechanisms of plant species diversity and plant functional diversity under warming and nitrogen deposition

The results of this study showed that the importance value of Grasses, the weighted mean of leaf nitrogen, the weighted mean of specific leaf area, the weighted mean of leaf area, and the weighted mean of leaf weight would have direct and indirect effects on plant species diversity and plant functional diversity through warming and nitrogen deposition. This may be because when temperature and nutrient limitations are alleviated, light resources often become an important factor in plant growth (Díaz et al. 2016). In this study area, the plant community under climate warming and nitrogen deposition is dominated by tall grasses and short herbs. Tall plants ( Poa pratensis and Elymus nutans ) usually have higher leaf area. A larger leaf area will increase the photosynthetic rate of plants, which gives tall plant species an advantage in light competition(Sun et al. 2022). Dwarf and sheltered plants are limited by light, and the species diversity and functional diversity of plant communities change accordingly (Tatarko and Knops 2018, Zheng et al. 2018).

In addition, the results of this study also showed that soil total nitrogen was the main factor affecting plant species diversity and plant functional diversity. In the study of temperate grasslands, it was also found that soil total nitrogen and soil nitrogen-phosphorus ratio were the most important variables to explain the plant functional diversity index (Rodríguez et al. 2023). This is because nitrogen is the main limiting resource for plant productivity and the main factor affecting the growth of grassland plants(Li et al. 2023). Due to differences in nutrient utilization strategies between plant species or functional groups, changes in soil nitrogen content may change the competitive relationship between different species in the plant community and the balance of nutrient requirements, resulting in changes in plant community composition and structure(Peñuelas et al. 2012, Mao et al. 2015). When the soil is rich in nitrogen, plants can more easily absorb enough nitrogen, which promotes their growth and reproduction. This will lead to the rapid growth and expansion of some plant species in the plant community, forming a competitive advantage, that may suppress the growth of other plants and reduce plant species diversity(Verma et al. 2015, Song et al. 2019). On the contrary, if the soil contains low levels of nitrogen, plants will face nitrogen limitation, resulting in slower growth and less competition, which may provide more plant species with the opportunity to survive, thereby increasing species diversity(Qu et al. 2022).

The results of this study also showed that soil C/N ratio was also an important factor affecting the species and functional diversity of plant communities. The study of 84 grasslands across six continents across a wide range of climatic gradients also found that plant diversity was positively correlated with soil C/N ratio(Spohn et al. 2023). This is mainly due to the low nutritional value of organic matter with a high carbon-nitrogen ratio to microorganisms and slow decomposition(Cotrufo et al. 2013). Relatively scarce nitrogen resources may affect the competition between plants, thus affecting the diversity of plant communities. At the same time, warming and nitrogen deposition will affect the species and functional diversity of plant communities, and the diversity of plant species increases the cost of soil microbial decomposition(Han et al. 2022). Very diverse organic compounds from different plant communities may decompose more slowly than less diverse organic matter from less diverse communities (El Moujahid et al. 2017, Lehmann et al. 2020). Soil factors determine which species or functional traits in plant communities will be retained, and this variability in plant species ' adaptation to the environment affects competition between communities and affects plant community structure(Wang et al. 2022b). Therefore, a better understanding of the relationship between plant community species diversity and soil physical and chemical properties will help to understand the overall function of grassland ecosystems better.

The results of this study also showed that with the increase of warming and nitrogen deposition, the plant species diversity index and plant functional diversity index decreased gradually, and there was a positive correlation between them. Some studies have also shown that the functional richness index is positively correlated with species richness(Bu et al. 2014, Ruiz-Benito et al. 2014), reflecting the occupation of niche space by existing species. The higher the richness, the more sufficient the niche space is occupied, the higher the community productivity, and the more stable the ecosystem function. This is mainly because warming and nitrogen deposition will reduce the niche dimension by alleviating resource constraints, which may aggravate species competition and lead to competitive exclusion, thereby reducing species diversity (Harpole et al. 2016, Hu et al. 2023). Recent studies have assessed changes in species richness from the perspective of functional traits and have shown that nitrogen-rich soils promote the growth of plant species with higher canopy and larger leaves, transforming underground nutrient competition into aboveground light competition (Hautier et al. 2009, Lavorel and Grigulis 2012, Harpole et al. 2016). The change of limiting factors is regarded as an indirect way to reduce the niche dimension, which leads to the reduction of functional diversity(Harpole et al. 2017).

**Question 8:You mention that warming increases plant height. Do you think this will have a knock-on effect on herbivory e.g. the taller species will be more targeted by herbivores and so actually be at a disadvantage under warming? My understanding is that herbivory plays an important role in the Qinghai-Tibet Plateau but it is not mentioned in the manuscript. A paragraph about the potential impacts of your findings on herbivores would be interesting.**

**Response:Thank you for your advice. In order to focus on the effects of climate warming and nitrogen deposition on plant community diversity, long-term fenced plots were selected in this study area, which also avoided the impact of livestock. Therefore, there is no mention of the influence of herbivores on plant community diversity. The influence of herbivores on plant community diversity will be the focus of our research group's next step. Thank you for your suggestions. I hope to have the opportunity to present this manuscript to you in the future.**

**Question 9: In the introduction you say “This study seeks to provide a theoretical basis for maintaining the species diversity and sustainability of plant communities in the alpine meadows of the Qinghai-Tibet Plateau under future climate warming and nitrogen deposition scenarios.”. However, I don’t see this mentioned in great detail in the discussion. It would be useful to have a paragraph about the implications of your results for future biodiversity in the Qinghai-Tibet Plateau and also any practical suggestions for how to maintain diversity in the future.**

**Response:Thank you for your advice. This part of the inquiry is the focus of this paper, and this edition does not mention any practical recommendations on the impact of the findings on the future biodiversity of the Tibetan Plateau, or how to maintain diversity in the future. The addition of this part makes the structure and content of this paper clearer and more in-depth. Thanks again for your suggestions, which have been added in the appropriate section of this article. Modified as follows (L709-L727) :**

**-**Conclusion

In the study of alpine meadows on the Qinghai-Tibet Plateau, both warming and nitrogen deposition had significant negative effects on the plant species diversity index and plant functional diversity index. Plant factors ( Grasses importance value, leaf nitrogen weighted mean, specific leaf area-weighted mean, leaf area-weighted mean, and leaf weight weighted mean ) and soil environmental factors ( soil total nitrogen and soil carbon-nitrogen ratio ) under warming and nitrogen deposition will directly or indirectly affect plant community diversity. At the same time, the relationship between plant species diversity and functional diversity in the alpine meadow of the Qinghai-Tibet Plateau gradually weakened with the increase of warming and nitrogen deposition. Combined with the above, the changes in plant species and functional diversity under climate warming and nitrogen deposition in the future may reduce the stability of the alpine meadow ecosystem, which is challenging for alpine meadow grassland restoration and ecosystem management. Therefore, regular monitoring of changes in plant species diversity and functional diversity will help to detect changes in alpine meadow ecosystems promptly and take measures to address biodiversity loss. At the same time, monitoring soil health status, especially soil carbon-nitrogen ratio, nitrogen content, and other indicators, is of great significance for taking corresponding land management measures.

**We believe that these revisions have significantly improved the manuscript and have addressed all the issues raised. Thank you again for your valuable feedback.**
